# Essential Oil from Vietnamese *Peperomia leptostachya* Hook. & Arn. (Piperaceae): Chemical Composition, Antioxidant, Anti-Inflammatory, Cytotoxic Activities, and In Silico Analysis

**DOI:** 10.3390/molecules29122808

**Published:** 2024-06-12

**Authors:** Hien Minh Nguyen, Ty Viet Pham, Hung Quoc Vo, Hoai Thi Nguyen, Linh Thuy Khanh Nguyen, Bao Chi Nguyen, Khanh Linh Chung, Duc Viet Ho

**Affiliations:** 1 Faculty of Pharmacy, Ton Duc Thang University, Ho Chi Minh City 700000, Vietnam; nguyenminhhien@tdtu.edu.vn (H.M.N.); chungkhanhlinh@tdtu.edu.vn (K.L.C.); 2 Faculty of Chemistry, University of Education, Hue University, 34 Le Loi, Hue 530000, Vietnam; 3 Faculty of Pharmacy, Hue University of Medicine and Pharmacy, Hue University, 06 Ngo Quyen, Hue 530000, Vietnam; vqhung@huemed-univ.edu.vn (H.Q.V.); nthoai@huemed-univ.edu.vn (H.T.N.); nktlinh@huemed-univ.edu.vn (L.T.K.N.); 4 Department of Science, Technology & International Relations, Hue University, 04 Le Loi, Hue 530000, Vietnam; ncbao@hueuni.edu.vn

**Keywords:** *Peperomia leptostachya*, essential oils, germacrene D, antioxidant, anti-inflammatory, cytotoxicity, in silico study

## Abstract

This study is the first to investigate the chemical composition and antioxidant, anti-inflammatory, and cytotoxic activities of *Peperomia leptostachya* leaf oil. A yellow oil was obtained through hydro-distillation, with a yield of 0.1% (*w*/*w*). The GC-MS analysis revealed 66 compounds, constituting 99.6% of the oil. Sesquiterpene hydrocarbons predominated (70.4%), followed by monoterpene hydrocarbons (13.2%), oxygenated sesquiterpenes (12.4%), non-terpenic compounds (2.0%), and oxygenated monoterpenes (1.6%). Major constituents included germacrene D (25.1%), (*E*)-caryophyllene (17.4%), bicyclogermacrene (6.6%), *α*-pinene (6.2%), and *β*-pinene (4.7%). The assessment of antioxidant capacity via 1,1-diphenyl-2-picrylhydrazyl (DPPH) scavenging assay yielded a weak effect, with an IC_50_ value > 100 µg/mL. The inhibition of lipopolysaccharide-induced nitric oxide production in RAW 264.7 cells was quantified using the MTT assay, showing an IC_50_ value of 15.15 ± 0.68 µg/mL. Furthermore, cytotoxic effects on SK-LU-1 cell line growth were evaluated using the sulforhodamine B assay, resulting in an IC_50_ value of 37.45 ± 2.43 μg/mL. The anti-inflammatory activity was notable among the analyzed bioactivities of this oil. By employing a computational model, the predominant secondary metabolites in the essential oil were selected as candidates for interaction analysis with cyclooxygenase-2, an enzyme implicated in the inflammatory response. Our findings suggest that *P. leptostachya* leaf oil could serve as a potential source of natural compounds with prospective therapeutic effects in treating inflammatory conditions.

## 1. Introduction

Essential oils (EOs) are a valuable source of plant-derived chemicals with antioxidant, anti-inflammatory, and cytotoxic properties [1]. The antioxidants in EOs reduce oxidative stress by neutralizing damaging radicals, thereby protecting cells and promoting overall health [2]. Additionally, EOs contain secondary metabolites such as monoterpenes, sesquiterpenes, and phenolic compounds, which modulate inflammation and immune response pathways, exhibiting significant anti-inflammatory effects [3]. Chronic inflammation and oxidative stress are two interrelated pathogenic processes contributing to cancer growth and progression [4]. Due to their remarkable biological and pharmacological effects, EOs play a valuable role in treating various diseases. Therefore, research to better understand the therapeutic potential of EOs is essential.

Among EOs from natural sources, the Piperaceae family, a large group within the order of Piperales, comprises over 4000 known species with cultural, medicinal, and economic importance [5,6]. Among the diverse genera in this family, *Peperomia* is a captivating group of flowering plants, with over 1600 known species distributed across tropical and subtropical regions [5,6]. The unique appearance of *Peperomia* plants, characterized by slender, erect stems and elongated, lance-shaped leaves with prominent venation, is highlighted for its ornamental appeal [7]. The *Peperomia* genus has a rich history of traditional use in various cultures for medicinal and therapeutic purposes. Many studies have highlighted the presence of alkaloids, flavonoids, terpenes, and phenolics in *Peperomia* species, which significantly contribute to these plant’s potential pharmacological effects [8]. Owing to these effects, *Peperomia* EOs have been used for wound healing and skin applications, gastrointestinal disorders, respiratory health, and antimicrobial and antifungal applications, as well as for their anti-inflammatory, analgesic, diuretic, and antihypertensive effects [9,10]. Due to the numerous medicinal properties and applications of this genus, the chemical composition of *Peperomia* Eos, such as *P*. *inaequalifolia*, *P. obtusifolia*, *P. pellucida*, *P. hernandiifolia*, *P. acuminata*, *P. galioides*, *P. chalhuapuquiana*, *P. macrostachyos*, *P. rotundifolia*, *P. circinnata*, and *P. serpens,* has been investigated in many studies. However, there are scarce studies on the chemical components and biological effects of *P. leptostachya* [10,11,12,13,14,15,16,17].

*Peperomia leptostachya* Hook. & Arn., also known as the slender peperomia or hairy peperomia, is a diminutive succulent herb. Previous research has been conducted on its unique and attractive appearance, growing habits, and distribution in tropical regions of Central and South America [18,19]. As a *Peperomia*, this species may hold the promise of potential bio-therapeutic properties. Exploring the chemical composition and biological activities of *P. leptostachya* is necessary to expand the knowledge about this species. The primary objective of this study was to identify the chemical composition and investigate the antioxidant, anti-inflammatory, and cytotoxic activities of *P. leptostachya* leaf EO. To the best of our knowledge, its components and biological properties were reported for the first time in this study. Furthermore, molecular docking was conducted to elucidate the active components responsible for the observed anti-inflammatory effects, with a primary emphasis on their capacity to inhibit the cyclooxygenase-2 (COX-2) enzyme, which plays a pivotal role in prostaglandin production.

## 2. Results

### 2.1. GC-MS Profiles

**Figure 1:**
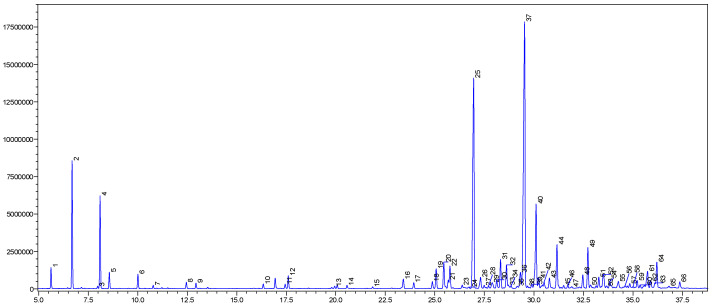
The GC chromatogram of *P. leptostachya* leaf EO.

The yellow oil of *P. leptostachya* leaves was obtained with a yield of 0.1% (*w*/*w*). The GC-MS analysis detected 66 compounds in the oil, representing 99.6% of its composition (Figure 1 and Table 1). Sesquiterpene hydrocarbon compounds were present in the highest amounts at 70.4%, followed by monoterpene hydrocarbons (13.2%), oxygenated sesquiterpenes (12.4%), non-terpenic compounds (2.0%), and oxygenated monoterpenes (1.6%). Germacrene D, (*E*)-caryophyllene, bicyclogermacrene, *α*-pinene, and *β*-pinene were revealed as the principal compounds in the oil, with percentages of 25.1, 17.4, 6.6, 6.2, and 4.7%, respectively. Some compounds, including *δ*-cadinene (3.1%), (*E*)-nerolidol (2.6%), *α*-humulene (1.9%), *β*-bourbonene (1.8%), valerianol (1.8%), *allo*-aromadendrene (1.6%), *epi*-*α*-cadinol (1.6%), *β*-elemene (1.5%), caryophyllene oxide (1.4%), *α*-copaene (1.3%), and *trans*-cadina-1(6),4-diene (1.3%), were detected in amounts greater than 1.0%.

### 2.2. Biological Activities of Essential Oil

Antioxidant Activity Detection Using 2,2-diphenyl-1-picrylhydrazyl (DPPH) and 2,2′-azino-bis(3-ethylbenzothiazoline-6-sulfonic Acid (ABTS) Scavenging Assays.

**Figure 2:**
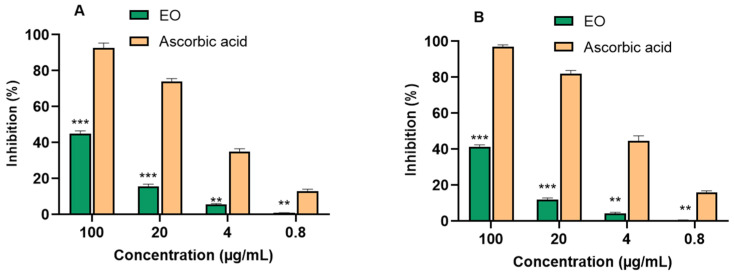
Free radical scavenging activity ((**A**) DPPH assay and (**B**) ABTS assay) from *P. leptostachya* leaf EO. ** *p* < 0.01, *** *p* < 0.001 compared with positive control.

The antioxidant activity of the *P. leptostachya* leaf EO was assessed using DPPH and ABTS assays. Overall, the results indicated that the EO did not exhibit any antioxidant properties, as its IC_50_ value exceeded 100 µg/mL compared with the positive control, ascorbic acid (IC_50_ 7.37 ± 0.27 µg/mL) for DPPH and (IC_50_ 4.58 ± 0.23 µg/mL) for ABTS assays (Figure 2 and Table 2).

The Anti-inflammatory Effects in LPS-Stimulated RAW 264.7 Macrophages.

**Figure 3:**
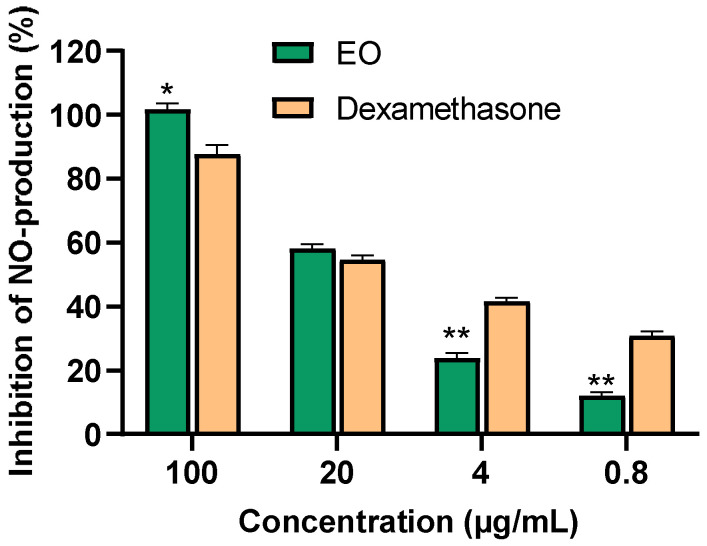
Inhibition of NO production (%) in LPS-stimulated RAW 264.7 cells of the *P. leptostachya* leaf EO. * *p* < 0.05, ** *p* < 0.01 compared with positive control.

In exploring the inhibitory effects of *P. leptostachya* leaf EO on lipopolysaccharide (LPS)-induced NO production in RAW 264.7 cells, the MTT assay revealed that *P. leptostachya* leaf EO displayed significant anti-inflammatory effects by inhibiting NO production, with an IC_50_ of 15.15 ± 0.68 µg/mL. This value closely resembled that of the positive control, dexamethasone, which had an IC_50_ of 12.61 ± 0.98 µg/mL. At a concentration of 20 µg/mL, the EO showed no cytotoxicity, with a cell survival rate of 87.06 ± 1.61% (Figure 3 and Table 2).

Cytotoxic Activity Against SK-LU-1 Cell Line.

**Figure 4:**
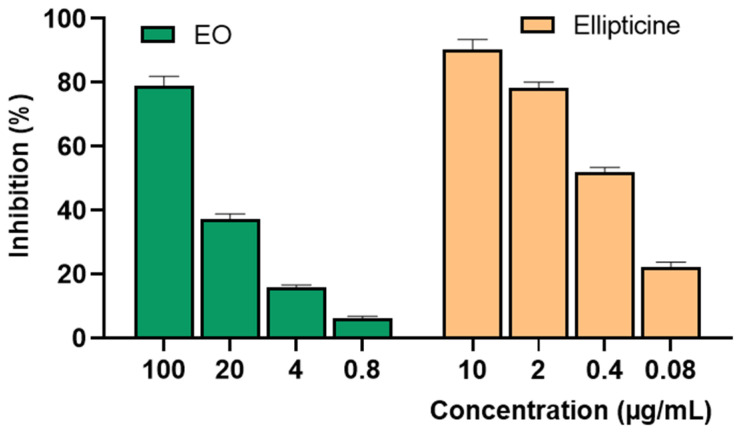
Inhibition of SK-LU-1 human cancer cell lines of *P. leptostachya* leaf EO.

Further investigation into the effect of *P. leptostachya* leaf oil on the SK-LU-1 cell line was performed using the sulforhodamine B (SRB) assay. The result indicated good cytotoxic effects, with an IC_50_ value of 37.45 ± 2.43 μg/mL, while the positive control, ellipticine showed highly significant cytotoxicity, with an IC_50_ value of 0.35 ± 0.04 µg/mL (Figure 4 and Table 2).

### 2.3. Molecular Docking Analysis

**Figure 5:**
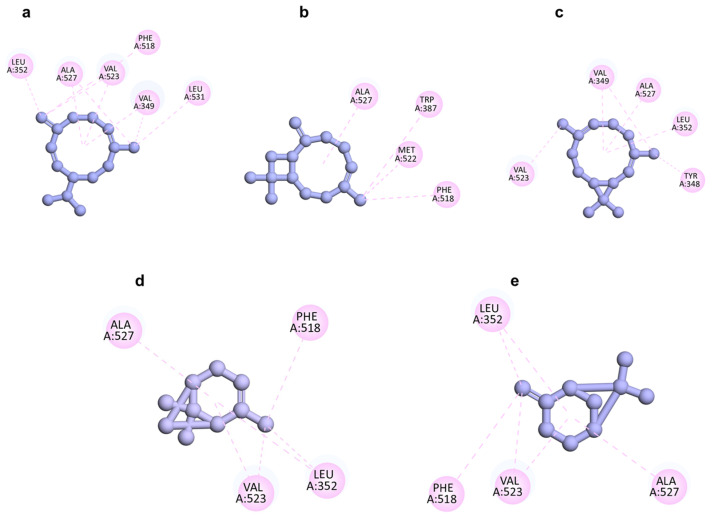
Two-dimensional docking poses showing interactions of germacrene D (**a**), (*E*)-caryophyllene (**b**), bicyclogermacrene (**c**), *α*-pinene (**d**), and *β*-pinene (**e**) in the binding sites of COX-2 (PDB ID: 1PXX).

In the computational investigation, a docking analysis was conducted to evaluate the interactions of the five predominant secondary metabolites extracted from *P. leptostachya* germacrene D (a), (*E*)-caryophyllene (b), bicyclogermacrene (c), *α*-pinene (d), and *β*-pinene (e), with the crystallographic structure of COX-2 complexed with diclofenac (PDB ID: 1PXX), retrieved from the Protein Data Bank. If a compound demonstrates strong binding affinity to the active site of the COX-2 enzyme, it suggests that the compound may inhibit the enzyme’s activity, thereby reducing inflammation. To validate the docking method, the cocrystallized ligand, diclofenac, was re-docked into the active site of the target protein, and the root-mean-square deviation (RMSD) between the re-docked pose and the original crystal structure was calculated. The obtained RMSD value of 1.890 fell within the acceptable range (RMSD ≤ 2.0 Å), indicating that our docking protocol was able to reproduce the experimental binding pose [21,22]. This validation confirmed the reliability of our docking results and supported the credibility of our in silico predictions (Figure 5).

**Figure 6:**
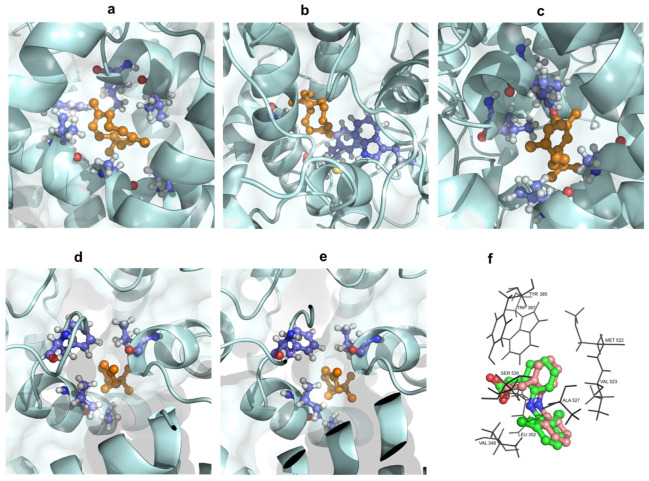
Three-dimensional docking poses showing interactions of germacrene D (**a**), (*E*)-caryophyllene (**b**), bicyclogermacrene (**c**), *α*-pinene (**d**), and *β*-pinene (**e**) in the binding sites of COX-2 (PDB ID: 1PXX); (**f**) Overlapped structures of native ligand crystal structure of diclofenac (green) and re-docked ligand (wheat).

A high binding energy indicates stronger interaction between the tested compounds and the protein. The five compounds, characterized by a lipophilic carbon framework, demonstrated moderate docking scores ranging from −8.6 to −6.0 kcal/mol compared with diclofenac as a co-ligand (−8.4 kcal/mol). Given their specific chemical structure, the interactions between these five docked compounds and the protein binding site primarily involved hydrophobic interactions. Residues Leu352, Val523, and Ala527 within the COX-2 binding pocket were identified as crucial contributors to this nonpolar interface formation. Among **a**–**e**, compound **a** exhibited the lowest docking score (−8.6 kcal/mol) against COX-2, which was comparable to the positive control diclofenac (−8.4 kcal/mol). It suggested that **a** had the strongest interaction with the COX-2 enzyme. Following this, **c** showed a docking score of −7.7 kcal/mol, followed by **b** (−6.6 kcal/mol), while **d** (−6.0 kcal/mol) was similar to **e** (−6.1 kcal/mol). In summary, the lowest docking scores observed for **a**–**e** correlated with the significant anti-inflammatory activity of the EO (Figure 5 and Figure 6 and Table 3). This suggested that these compounds may play a key role in mediating the anti-inflammatory effects of *P. leptostachya* leaf EO by interacting strongly with the COX-2 enzyme, thus prompting further investigation into their therapeutic potential.

Statistical Analysis: The results were expressed as mean ± standard deviation (SD), *n* = 3. The results were analyzed using GraphPad Prism software (version 9.3.0, GraphPad Software, San Diego, CA, USA) and Microsoft Excel software (Excel 2010 version, Redmond, Washington, DC, USA). Statistical differences were determined busing *t*-Student test with * *p* < 0.05; ** *p* < 0.01; *** *p* < 0.001.

## 3. Discussion

The chemical compositions of EOs from various species of *Peperomia* had previously been explored. In the following, we compare and contrast the chemical compositions of EOs among different *Peperomia* species. To date, the EOs from various *Peperomia* species have exhibited diverse chemical profiles. *P. inaequalifolia* from Quito, Ecuador, contains significant amounts of safrole and 11αH-himachal-4-en-1β-ol [23], while in specimens from Guayllabamba parish, elemicin and α-bisabolol predominate [10]. Similarly, *P. obtusifolia* EOs are dominated by *β*-caryophyllene in Pakistan and valerianol in Brazil [11,24]. *P. pellucida* features linalool and limonene in southwestern Nigeria and *β*-fernesene in Kwara State [12,13]. Dillapiole predominates in Brazilian *P. pellucida* [14]. *P. acuminata* EOs from Venezuela primarily contain (2*E*)-dodecenal [15]. *P. borbonensis* from Réunion Island contains myristicin and elemicin [16]. Investigating the volatile oil composition of fresh and dried aerial parts of *P. galioides* and *P. chalhuapuquiana* reveals different components [18]. *P. galioides* EO has also been found to contain globulol and *β*-caryophyllene [25], while *P. macrostachya* contains epi-α-bisabolol and caryophyllene oxide [13]. *P. rotundifolia* is rich in decanal and dihydro-*β*-santalol [14]. *P. circinnata* EOs from the Amazon contain myrcene and *β*-phellandrene [26], while those from the Pará state contain myrcene and *β*-phellandrene [27]. In addition, the EO of *P. serpens* features (*E*)-nerolidol and ledol [28]. We addressed the slight differences in retention indices between our study and the literature. These differences can be attributed to several factors, including instrumental variability, differences in experimental conditions, sample preparation methods, and intrinsic variability of the compounds. Instrumental variability involves differences in GC-MS systems and columns, such as column dimensions and stationary phases, which can affect retention times. Variations in experimental conditions, like temperature programming and carrier gas flow rates, also play a significant role. Additionally, differences in sample preparation techniques, such as extraction methods and solvent purity, can lead to changes in the chemical composition and relative abundance of compounds. Furthermore, the chemical components may result in variability in their interactions with the column’s stationary phase, impacting retention indices. Our data analysis revealed that *β*-caryophyllene predominates as the main compound in EOs derived from numerous species within *Peperomia*. However, it was noteworthy that germacrene D was the primary compound detected in the EO of *P. leptostachya* in this study, which distinguished it from other species within the genus. The presence of germacrene D in significant amounts in *P. leptostachya* EO, while scarce or undetected in other species, suggests a species-specific chemical profile influenced by environmental factors, geographical location, and the inherent characteristics of each species. Furthermore, there is limited research exploring the biological effects of *Peperomia* EOs, including their antioxidant, anti-inflammatory, and cytotoxic properties. For this reason, we investigated the biological activity of the leaf oil of *P. leptostachya*.

We first assessed the antioxidant capability of *P. leptostachya* leaf oil using the DPPH scavenging assay. Previous investigations have focused on the antioxidative activity of EOs derived from *Peperomia*, including for *P. inaequalifolia*, *P. pellucida*, and *P. inaequalifolia*. Assessments utilizing the DPPH, ABTS assays and antioxidant activity studies revealed noteworthy activity in these EOs. The ABTS assay outperformed the DPPH assay in accurately assessing the antioxidant levels of samples containing hydrophilic and lipophilic components. However, the present study found no antioxidant activity in *P. leptostachya* leaf EO, which showed no activity. The absence of phenolic compounds detected in this study may contribute to this EO’s relatively low antioxidant activities.

EOs have been widely used in traditional medicine to treat respiratory illnesses through inhalation and oral administration. When ingested, essential oils are absorbed through the digestive system into the bloodstream and distributed throughout the body, including in the lungs [29]. Lung cancer is one of the most common diseases worldwide, affecting millions of people each year and acting as a leading cause of cancer-related deaths globally [30]. Therefore, we investigated the inhibitory effects of *P. leptostachya* leaf oil on the SK-LU-1 cell line using the SRB assay. The key components in *P. leptostachya* leaf oil contributed to its substantial cytotoxic activity, with an IC_50_ value of 37.45 ± 2.43 µg/mL. Additionally, previous studies suggested that (*E*)-caryophyllene and germacrene D exhibited notable cytotoxic effects against a range of tumor cell lines, which supports our findings.

Research on the anti-inflammatory activity of EOs from the genus *Peperomia* was limited. Currently, there is only one study on the anti-inflammatory activity of *P. serpens,* indicating a significant anti-inflammatory effect in acute inflammation models induced by paw edema from carrageenan, dextran, and croton oil, as well as leukocyte and neutrophil migration into the peritoneal cavity in mice. Additionally, real-time microscopic analysis showed reduced neutrophil rolling and adhesion in the mesenteric microcirculation of mice [28]. Of the three bioactivities tested in the present study, the anti-inflammatory effect of the EO from the plant was particularly remarkable (IC_50_ 15.15 ± 0.68 µg/mL). Docking models were employed to analyze the potential interactions with the target protein of the bioactive compounds. Cyclooxygenase-1 (COX-1) and COX-2 are enzymes involved in the synthesis of prostaglandins, lipid compounds with diverse physiological effects. While COX-1 is constitutively expressed in many tissues and contributes to maintaining normal physiological functions, such as gastric mucosa protection, platelet aggregation promotion, and renal blood flow regulation, COX-2 is typically absent in most tissues but is primarily responsible for increased prostaglandin production during inflammation. Due to its role in increased prostaglandin production, COX-2 was selected as the target for this study [31,32]. The anti-inflammatory activity of *P. leptostachya* may be attributed to the binding affinity of the majority compounds (e.g., germacrene D, (*E*)-caryophyllene, bicyclogermacrene, *α*-pinene, and *β*-pinene) present in the EOs, highlighting the potential therapeutic significance of these compounds in modulating inflammatory processes and warranting further investigation into their mechanisms of action and clinical applications.

It is important to recognize that the inhibitory properties of the EO cannot only be attributed to its primary components. The anti-inflammatory efficiency might result from additive, synergistic, or antagonistic effects of the EO constituents [33]. Notably, even less common and minor chemicals in trace amounts (<0.5%) may significantly affect the EO’s biological activity. For instance, compounds such as sabinene, linalool, (*Z*)-cinnamyl alcohol, *β*-oplopenone, and *β*-selinene, though present in minute quantities, have been found to play pivotal roles in the anti-inflammatory activity of EOs [34,35,36]. Understanding the synergistic interactions among these compounds, including the minor constituents, is paramount for comprehending the biological effectiveness of the EOs. Another limitation of our study is the unclear mechanism of action underlying the anti-inflammatory effects of *P. leptostachya* EO. Inflammation can manifest through various pathways. However, the investigation primarily focused on conducting docking simulations to analyze the interaction between the major components of the extracted EO and COX-2, a target protein associated with inflammation inhibition. Therefore, further investigations are needed to specify the signaling pathways in the anti-inflammatory process.

## 4. Materials and Methods

### 4.1. General Procedures

The bioactivity measurements were conducted using a BioTex El800 UV-Vis spectrophotometer (Boston, MA, USA). The positive controls and reagents were obtained from Sigma Aldrich (Saint Louis, MO, USA). Dulbecco’s Modified Eagle’s Medium (DMEM) and fetal bovine serum (FBS) were sourced from Life Technologies, Inc. (Gaithersburg, MD, USA). The RAW 264.7 cells were provided by Prof. Domenico Delfino, University of Perugia, Italy.

### 4.2. Plant Material and Extraction

On 7 July 2023, fresh leaves of *P. leptostachya* were gathered from Quangtri, Vietnam, situated at coordinates 16°46′56.3″ N 106°35′03.9″ E. Dr. Anh Tuan Le identified the botanical name of the plant. A voucher specimen labeled PLQT-2023 (*P. leptostachya* leaves) was deposited at the Faculty of Chemistry, University of Education, Hue University in Vietnam.

### 4.3. Distillation

Each fresh powder sample weighing 1.0 kg underwent hydro-distillation using a Clevenger apparatus. The extraction process was conducted for 4.0 h, facilitating the release of EOs from the plant material. Following hydro-distillation, the oil was obtained by decantation and subsequently dried over Na_2_SO_4_ to eliminate any residual water content. The resulting dried EO was then stored in sealed vials under a temperature of −5 °C to safeguard their stability and maintain the integrity of its chemical composition for subsequent analysis [37].

### 4.4. GC-MS Analysis

The GC-MS analysis was conducted using a Shimadzu Technologies GCMS-QP2010 Plus chromatograph (Shimadzu, Kyoto, Japan), equipped with a fused silica Equity-5 capillary column (30 m × 0.25 mm, film thickness 0.25 µm, Supelco, Bellefonte, PA, USA) [38,39,40,41]. The analytical parameters were set as follows: helium was utilized as the carrier gas at a flow rate of 1.2 mL/min, while the injector and interface temperatures were maintained at 280 °C. The temperature program entailed a ramp from 60 °C (held for 3 min) to 240 °C (held for 15 min) at a rate of 3 °C/min, followed by a final ramp to 280 °C (held for 35 min) at a rate of 5 °C/min for the column. Sample injection utilized a split ratio of 15:1, with an inlet pressure of 93.2 kPa and an injection volume of 1.0 µL. The mass spectrometer settings included an ionization voltage of 70 eV, a detector voltage of 0.82 kV, and data acquisition in the scan mass range of 50–500 amu at a sampling rate of 0.5 scan/s. Identification of chemical constituents was achieved by co-injecting the samples and comparing the retention indices (RIs) to a homologous series of *n*-alkanes (C_7_–C_40_), as well as by referencing the Adams book [20]. Quantification was carried out based on the relative peak area percentage. Comprehensive analysis of the obtained GC-MS data facilitated the identification and characterization of the chemical compounds present in the *P. leptostachya* leaf EO, laying the groundwork for further investigations and potential applications.

### 4.5. DPPH and ABTS Radical Scavenging Activities

The assessment of *P. leptostachya* leaf-derived EO’s capacity to neutralize free radicals generated from DPPH and ABTS adhered to the methodology outlined, with suitable modifications implemented to align with laboratory conditions. The specific protocols for these evaluations were detailed in our prior reports [42,43].

### 4.6. Anti-Inflammatory Assay

The inhibitory activity of *P. leptostachya* leaf EO against LPS-induced nitric oxide (NO) production in RAW 264.7 cells was evaluated. The concentration of nitrite, indicating NO presence in the culture medium, was assessed using the Griess reaction. The detailed protocols for these assessments were outlined in our previous reports [42].

### 4.7. Cytotoxicity Assays

The cytotoxicity of the EO from *P. leptostachya* leaf was assessed using a sulforhodamine B assay on the SK-LU-1 cell line. Our earlier study described the detailed protocols for these experiments, focusing on cytotoxicity [44].

### 4.8. In Silico Analysis

The molecular docking analyses were conducted using AutoDock Tools 1.5.7 and AutoDock Vina 1.1.2. The crystal structure of cyclooxygenase-2 (PDB ID: 1PXX) was retrieved from the Protein Data Bank (PDB). Visualization of the compound–protein interactions was performed using BIOVA Discovery Studio Visualizer v24.1.0.23298 [45].

## 5. Conclusions

Our research provides initial insights into the chemical composition and biological characteristics of *P. leptostachya* leaf oil, marking its first investigation. Through GC-MS analysis, we identified 66 compounds, with germacrene D emerging as the predominant compound, comprising 25.1% of the oil. The chemical composition of *P. leptostachya* EO differs significantly from that of the other *Peperomia* species. Our biological assessments revealed weak antioxidant activity, significant anti-inflammatory effects against NO production in RAW 264.7 cells, and good cytotoxicity against SK-LU-1 cells. The prevalence of five main compounds supported the significant anti-inflammatory impacts of the EOs. These compounds showed potential to interact with COX-2 and mitigate the production of pro-inflammatory prostaglandins. Overall, *P. leptostachya* leaf EO showed prospective anti-inflammatory and anti-cancer properties, the molecular mechanisms of which should be investigated in future work.

## Figures and Tables

**Table 1:** Chemical compounds in *P. leptostachya* leaf EO.

No	RT	Compound	RI Exp	RI Lit	Concentration (%)	Classify Compds
1	5.62	*n*-Nonane	900	900	0.9	-
2	6.69	*α*-Pinene	933	932	6.2	MH
3	7.97	Sabinene	972	969	0.1	MH
4	8.10	*β*-Pinene	976	974	4.7	MH
5	8.57	*β*-Myrcene	991	988	0.8	MH
6	10.01	Limonene	1028	1024	0.8	MH
7	10.78	(*E*)-*β*-Ocimene	1047	1044	0.2	MH
8	12.45	Terpinolene	1088	1086	0.4	MH
9	12.94	Linalool	1100	1095	0.3	OM
10	16.34	Terpinen-4-ol	1177	1174	0.3	OM
11	16.94	*α*-Terpineol	1191	1186	0.7	OM
12	17.60	*n*-Decanal	1206	1201	0.8	-
13	19.94	(*Z*)-Cinnamyl alcohol	1259	1259	0.1	-
14	20.56	*n*-Decanol	1273	1272	0.2	-
15	21.86	Carvacrol	1302	1298	0.1	OM
16	23.41	*δ*-Elemene	1338	1335	0.7	SH
17	23.93	*α*-Cubebene	1350	1348	0.4	SH
18	24.87	*α*-Ylangene	1372	1373	0.5	SH
19	25.07	*α*-Copaene	1377	1374	1.3	SH
20	25.45	*β*-Bourbonene	1386	1387	1.8	SH
21	25.68	*iso*-Longifolene	1391	1389	0.6	SH
22	25.76	*β*-Elemene	1393	1389	1.5	SH
23	26.38	(*Z*)-Caryophyllene	1408	1408	0.2	SH
24	26.71	*cis*-Nerone	1416	1412	0.2	OM
25	26.96	(*E*)-Caryophyllene	1422	1417	17.4	SH
26	27.31	*β*-Copaene	1430	1430	0.9	SH
27	27.49	*γ*-Elemene	1435	1434	0.2	SH
28	27.71	Aromadendrene	1440	1439	0.1	SH
29	27.89	6,9-Guaiadiene	1444	1442	0.5	SH
30	28.12	Spirolepechinene	1450	1449	0.6	SH
31	28.32	*α*-Humulene	1455	1452	1.9	SH
32	28.62	*allo*-Aromadendrene	1462	1458	1.6	SH
33	28.71	*cis*-Cadina-1(6),4-diene	1464	1461	0.2	SH
34	28.85	*cis*-Muurola-4(14),5-diene	1468	1465	0.2	SH
35	29.16	Dauca-5,8-diene	1475	1471	0.1	SH
36	29.32	*trans*-Cadina-1(6),4-diene	1479	1475	1.3	SH
37	29.53	Germacrene D	1484	1480	25.1	SH
38	29.68	*β*-Selinene	1488	1489	0.1	SH
39	29.89	*cis*-*β*-Guaiene	1493	1492	0.2	SH
40	30.11	Bicyclogermacrene	1498	1500	6.6	SH
41	30.24	*α*-Muurolene	1501	1500	0.7	SH
42	30.52	*δ*-Amorphene	1509	1511	0.4	SH
43	30.78	*γ*-Cadinene	1515	1513	0.7	SH
44	31.16	*δ*-Cadinene	1525	1522	3.1	SH
45	31.50	*γ*-Cuprenene	1534	1532	0.2	SH
46	31.71	*α*-Cadinene	1539	1537	0.2	SH
47	31.91	*α*-Calacorene	1544	1544	0.2	SH
48	32.47	Germacrene B	1558	1559	0.9	SH
49	32.73	(*E*)-Nerolidol	1565	1561	2.6	OS
50	32.86	Maaliol	1568	1566	0.2	OS
51	33.28	Spathulenol	1579	1577	0.7	OS
52	33.50	Caryophyllene oxide	1585	1582	1.4	OS
53	33.67	*cis*-*β*-Elemenone	1589	1589	0.1	OS
54	33.83	Viridiflorol	1593	1592	0.5	OS
55	34.27	Ledol	1605	1602	0.5	OS
56	34.60	*β*-Oplopenone	1614	1607	0.1	OS
57	34.84	Humulane-1,6-dien-3-ol	1620	1619	0.3	OS
58	34.84	trans-Isolongifolanone	1625	1625	0.3	OS
59	35.22	1-*epi*-Cubenol	1630	1627	0.5	OS
60	35.36	*cis*-Cadin-4-en-7-ol	1634	1635	0.2	OS
61	35.74	*epi*-*α*-Cadinol	1644	1638	1.6	OS
62	35.89	*α*-Muurolol	1648	1644	0.5	OS
63	36.11	*α*-Cadinol	1654	1652	0.4	OS
64	36.21	Valerianol	1657	1656	1.8	OS
65	36.82	Bulnesol	1673	1670	0.2	OS
66	37.36	Eudesma-4(15),7-dien-1*β*-ol	1688	1687	0.5	OS
		Total			99.6	
		Monoterpenes (MH)			13.2	
		Monoterpenoids (OM)			1.6	
		Sesquiterpenes (SH)			70.4	
		Sesquiterpenoids (OS)			12.4	
		Other compounds (-)			2.0	

RT: retention time, RI_E_: retention indices relative to *n*-alkanes (C_7_–C_40_) on Equity-5 column, RI_L_: retention indices from the Adams book [20], (-) means other compounds.

**Table 2:** IC_50_ values of *P. leptostachya* leaf EO.

The Half-Maximal Inhibitory Concentration: IC_50_ Values (µg/mL)				
Sample	Antioxidant Activity		Anti-Inflammatory Activity	Cytotoxic Activity
	DPPH	ABTS		
EOs	>100	>100	15.15 ± 0.68	37.45 ± 2.43
Positive controls	7.37 ± 0.27 ^a^	4.58 ± 0.23 ^a^	12.61 ± 0.98 ^b^	0.35 ± 0.04 ^c^

^a^ Ascorbic acid; ^b^ Dexamethasone; ^c^ Ellipticine.

**Table 3:** Docking scores (kcal/mol) of predominant constituents from *P. leptostachya* leaf EO.

Compound	Docking Score (kcal/mol)	The Concentration in the EO (%)
a. Germacrene D	−8.6	25.1
b. (*E*)-caryophyllene	−6.6	17.4
c. Bicyclogermacrene	−7.7	6.6
d*. α*-pinene	−6.1	6.2
e*. β*-pinene	−6.0	4.7
Positive control: Diclofenac	−8.4	

## Data Availability

The original contributions presented in the study are included in the article, further inquiries can be directed to the corresponding authors.
